# Does the Digital Economy Promote the Reduction of Urban Carbon Emission Intensity?

**DOI:** 10.3390/ijerph20043680

**Published:** 2023-02-19

**Authors:** Shouwu Jing, Feijie Wu, Enyi Shi, Xinhui Wu, Minzhe Du

**Affiliations:** 1 School of International Trade, Shanxi University of Finance and Economics, Taiyuan 030006, China; 2 School of Economics and Management, South China Normal University, Guangzhou 510006, China

**Keywords:** digital economy, carbon emission intensity, human capital investment, green innovation, two-way fixed effect model

## Abstract

The impact of the digital economy is increasing, and its environmental effect has attracted more and more attention. The digital economy promotes the improvement of production efficiency and the government’s environmental governance capacity, and contributes to the reduction of urban carbon emission intensity. In order to study the impact of digital economy development on urban carbon emission intensity, this paper analyzes the theoretical basis of the digital economy on the reduction of carbon emission intensity, and then, based on the panel data of cities from 2011 to 2019, uses the two-way fixed effect model for empirical testing. The regression results show that the development of the digital economy has promoted the reduction of carbon emission intensity of cities, promoted the green transformation and upgrading of cities, and lays a foundation for China to achieve carbon peaking and carbon neutralization through the improvement of human capital investment and green innovation level. The basic conclusion is robust by changing core explanatory variables, changing samples, replacing regression methods, and shrinking and truncating tests. The impact of the digital economy on urban carbon emission intensity varies with the location, grade and size of the city. Specifically, the development of the digital economy in cities in the eastern and central regions, cities at or above the sub provincial level, large cities and non-resource-based cities has promoted the reduction of urban carbon emission intensity. In terms of resource-based cities, the development of the digital economy in renewable resource-based cities and resource-based cities dominated by iron ore and oil mining has promoted the decline in urban carbon emission reduction intensity.

## 1. Introduction

The digital economy is a digital transformation in the process of economic development based on digital technologies, including 5G, artificial intelligence, big data, etc. The digital economy has a bearing on the overall development of a country, becoming an important field for countries around the world to seize the commanding heights of economic and social development, and has a profound impact on global development [1,2,3]. In 2020, the scale of China’s digital economy was 39.2 trillion yuan, accounting for 38.60% of GDP. From 2016 to 2020, the total volume of China’s digital economy increased by 1.74 times, with an annual average growth rate of 17.08%. The growth rate of the digital economy is much higher than that of GDP in the same period. As far as the development of urban digital economy, a development pattern has taken shape overall, with “Beijing, Shanghai, Guangzhou, Shenzhen and Hangzhou” as the lead and provincial capitals as the main part. Beijing, Shanghai and Hangzhou are the top three cities in the overall ranking of the digital economy index. Cities in the eastern region perform better in terms of digital economy policy and environment, scale and quality of the digital economy. Guiyang performed better in digital economy policy and environment, while Chengdu, Chongqing and Xi’an ranked better in digital economy scale and quality. The development of China’s digital economy has made great progress. The digital economy can effectively support sustainable economic development [4], which has become a new driving force for China’s high-quality economic development [5,6]. Green development is an important part of high-quality economic development., and achieving carbon peak and carbon neutralization is also an important starting point for green economic development. Governments around the world have taken many steps to reduce CO_2_ emissions [7,8,9,10,11].

In terms of carbon emissions, China’s carbon emissions increased from 8145.8 million tons in 2010 to 9899.3 million tons in 2020, with an average annual growth rate of 2.4%. In 2021, China’s carbon emissions accounted for 31.116% of the world’s total carbon emissions [12]. China is the largest emitter of carbon dioxide in the world [13]. The rapid growth of “volume” and “speed” of carbon emissions makes the Chinese government face huge pressure to reduce emissions in the international community. However, the Chinese government attaches great importance to the climate and ecological environment changes caused by carbon emissions, and China proposed to achieve carbon peaking by 2030 and carbon neutrality by 2060 at the General debate of the 75th session of the UN General Assembly in 2020. All countries in the world regard low-carbon green development as an important means to maintain competitive advantage. Low carbon development is not only a key link in China’s transformation and upgrading, but also an important focus for China to participate in world competition.

The digital economy has significantly changed the production and organization form. We would wonder whether the development of the digital economy could have a positive impact on the green development of the economy? Can the development of the digital economy effectively promote the reduction of carbon emission intensity? If the development of the digital economy effectively reduces the intensity of carbon emissions, what are the mechanisms behind it? Could the effect of reducing carbon emission intensity of the digital economy have different results depending on the region, size, level and resource dependence of the city? Although many scholars have studied the economic and social development effects brought by the digital economy and the influencing factors of carbon emissions, respectively, there is a lack of detailed research on the carbon emission intensity of the digital economy. The answers to the above questions can further clarify the impact of digital economy development on China’s carbon emission intensity and the direction of China’s carbon emission reduction efforts, and provide an important reference for China’s economic development and environmental protection.

In the face of the increasingly serious energy crisis, global climate deterioration, ecological environment damage, and the pressure of China’s economic transformation and upgrading, it is of great significance to study the impact of the digital economy on urban carbon emission intensity, whether the development of the digital economy can achieve ecological environment protection. Research has shown that the digital economy can effectively promote economic transformation and upgrading [14], but can the digital economy reduce carbon emission intensity, and how? There is no clear answer. Therefore, this paper analyzes the theoretical mechanism of the digital economy to reduce carbon emission intensity, and based on the panel data of cities from 2011 to 2019, uses the two-way fixed effect model to empirically test the impact, mechanism and heterogeneity of digital economy development on urban carbon emission intensity. The research results show that first, the development of the digital economy has significantly reduced the intensity of urban carbon emissions. Second, the digital economy reduces the intensity of urban carbon emissions by increasing the level of human capital investment and improving the level of green innovation. Third, the digital economy has a heterogeneous effect on the reduction of urban carbon emission intensity. Specifically, the development of the digital economy has a significant effect on the reduction of carbon emission intensity of cities in the eastern and central regions, cities above the sub provincial level, large cities and non-resource-based cities. For resource-based cities, it is mainly renewable resource-based cities and resource-based cities dominated by iron ore and oil that have achieved a reduction in carbon emission intensity.

The possible marginal contributions of this paper are as follows. First, most of the existing research literatures do not link the development of the digital economy with carbon emissions. More attention is focused on the scale measurement of the digital economy and its impact on economic transformation and upgrading, and less on the impact of the development of the digital economy on the intensity of carbon emissions. Therefore, this paper makes up for this shortcoming and analyzes the impact of the development of the digital economy on carbon emission intensity from the urban level. Second, most of the existing literatures focus on the research on the measurement of digital economy development level and carbon emissions at the national and provincial levels. This paper analyzes from a more microscopic perspective, that is, the urban level, and further finds the difference of the effect of the digital economy on the reduction of carbon emission intensity in different heterogeneous cities. Third, this paper examines the mechanism of reducing urban carbon emission intensity by the digital economy. Specifically, the digital economy can reduce the intensity of urban carbon emissions by improving the level of human capital investment and the level of green innovation, and can be more targeted and flexible in policy formulation through the identification effect mechanism.

The rest of this paper is arranged as follows. Section 2 contains the literature review and theoretical basis. Section 3 contains the variables and model selection. Section 4 contains the basic regression analysis, including basic regression results, robustness test and endogenous test. Section 5 contains the mechanism test. Section 6 contains further analysis. Section 7 contains the conclusions and policy recommendations.

## 2. Literature Review and Theoretical Basis

### 2.1. Literature Review

The literature review part analyzes the impact of the digital economy on economic development and the factors affecting carbon emissions.

#### 2.1.1. The Impact of Digital Economy on Economic Development

The role of the digital economy in economic and social development is becoming more and more obvious. Countries around the world have taken improving the development level of the digital economy as the commanding height of future world competition. The digital economy can effectively promote economic development [14]. It can not only expand the economic scale, but also promote high-quality economic development.

From a macro perspective, the digital economy promotes high-quality economic development by improving the quality of research and development, upgrading the industrial structure and improving total factor productivity. The digital economy can effectively improve the quality and efficiency of R&D and promote high-quality economic development. On the one hand, it can reduce the restrictive impact of time and space on the interaction of R&D personnel, on the other hand, it can promote the shortening of R&D cycle and improve the development efficiency of new products and technologies [15]. Additionally, the digital economy can realize the upgrading effect of industrial structure and promote high-quality economic development. It can effectively promote the coordination between product production departments and realize digital manufacturing. Wallace [16] believes that the digital economy could unleash the potential of economic growth and promote inclusive economic development. Moreover, the digital economy improves total factor productivity and promotes high-quality economic development. The improvement of total factor productivity is the key to the long-term stable development of China’s economy and the successful transformation of China’s economy [17,18]. It can improve total factor productivity [19,20], but there are regional differences in its role. As the research of Yang and Lu [21] shows, the productivity level is low and the total factor productivity in the central and western regions has more room to improve. Therefore, the development of the digital economy plays a more significant role in improving the productivity level and the total factor productivity in the central and western regions.

From the micro perspective, the digital economy can improve the decision-making level of enterprise managers, improve the input-output efficiency and improve the level of entrepreneurship, and promote high-quality economic development. The digital economy promotes the improvement of enterprise management, improves organizational performance [22], and thus improves the level of high-quality economic development. Jiao [23], taking JingDong as an example, found that the digital economy has promoted the reorganization of JingDong group’s capabilities in terms of opportunity grasping, structural change, etc., and has embedded the most important data elements in the digital economy into the entire process of enterprise operation, achieving significant improvement in digital capabilities. Therefore, the digital economy can effectively promote the growth of enterprises, and further promote the long-term economic growth and structural upgrading. Meanwhile, the development of the digital economy can also effectively improve the input-output efficiency of enterprises and improve the quality of economic development. The digital transformation of enterprises not only changes the original organizational structure and governance structure of enterprises [24], but also improves the communication efficiency of enterprises, reduces resource redundancy, makes it more in line with the needs of marketization, and further improves the input and output efficiency of enterprises [25]. Moreover, the digital economy has further improved the quality of economic development by promoting the quality of entrepreneurship. The development of the digital economy has promoted the development of entrepreneurship ecosystem [26], which is more conducive to entrepreneurship [27]. On the one hand, it has accelerated the withdrawal of low-quality entrepreneurship from the market, on the other hand, it has promoted the entrepreneurship of general industry workers [28]. For rural residents, the digital economy has also improved the entrepreneurial environment and increased entrepreneurial opportunities, provided rural areas with opportunities to take advantage of digital technologies [29], activated entrepreneurial activities of rural residents [30], driven economic development in rural areas, and narrowed the urban-rural gap.

However, while the digital economy plays a positive role, it may also have negative effects. Syed et al. [31] takes digital finance as an example and believes that digital finance may also produce systemic risks and lead to the reduction of financial stability. Rochet and Tirole [32] believe that the digital economy may cause the contraction of traditional economy, reducing economic benefits.

#### 2.1.2. Influential Factors of Carbon Emissions

Global warming affects economic and social development [33], so low-carbon economic development is crucial. The existing literature has explored the factors affecting carbon emissions, and the impact of macro carbon emission reduction policies on carbon emissions. Administrative carbon emission reduction policies mainly include environmental regulations and environmental goal constraints [34,35]. Administrative carbon emission reduction policies can promote the reduction of carbon emissions and carbon emission intensity. However, at the same time, it may also cause a distortion in resource allocation and a decline in welfare. Strict environmental regulations may change producers’ expectations, increase carbon emissions in the short term [36], and even lead to pollution migration [37]. Furthermore, the impact of market mechanism on carbon emissions is noteworthy; it is generally believed that market mechanism can effectively reduce resource distortion, reduce energy consumption intensity and reduce carbon emissions. Tang et al. [38] found that carbon trading in pilot areas significantly promoted the reduction of carbon emission level. Higher levels of human capital and technology transfer from developed countries can contribute to the decline in CO_2_ emissions [39].

### 2.2. Theoretical Analysis and Research Hypothesis

The digital economy is the fourth economic form after agricultural economy, industrial economy and information economy. It is a new economic form developed mainly by using big data, artificial intelligence, the Internet of Things, 5G and others [15,40]. First, the development of the digital economy has promoted the improvement of production efficiency, which not only improves the labor production efficiency, but also improves the green total factor productivity, thereby reducing the intensity of urban carbon emissions. The development of the digital economy has promoted the increase in urban output, especially the increase in high-quality output, and reduced the emission level of pollutants. Specifically, the digital economy promotes the coordinated development of various elements, realizes the full utilization of resources, improves the efficiency of resource allocation, improves the efficiency of energy resource utilization, reduces the resource redundancy of the main factor users, reduces the level of pollution emissions, and promotes the reduction of urban carbon emission intensity. At the same time, the digital economy has promoted the upgrading of urban industrial structure through the use of new technologies and new means, moving from the traditional industrial structure relying on high input, low output and high pollution emissions to high-end manufacturing, and realizing the coordinated development and mutual integration of manufacturing and modern service industries. The intensity of urban carbon emissions has been further reduced through the upgrading of industrial structure. In addition, with the in-depth development of the digital economy, data has become an important strategic asset. At the same time, China has accelerated the promotion of the national strategy of big data and included data as an emerging factor in the factor marketization reform. The development of the digital economy has greatly promoted the positive role of data as a factor of production in promoting enterprises’ total factor productivity and even green total factor productivity. The development of the digital economy not only improves the information transparency of the use of data elements, but also improves the organizational and operational performance of enterprises through the use of big data. The development of the digital economy integrates land, labor, capital, technology and data in a coordinated manner, which increases the output of enterprises and reduces carbon emissions, reducing the intensity of carbon emissions.

Second, the development of the digital economy has improved the government’s governance ability, especially the environmental governance ability, and promoted the reduction of urban carbon emission intensity. On the one hand, the digital economy has improved the level of government governance and reduced the intensity of urban carbon emissions. The improvement of the government’s environmental governance level can not only effectively reduce haze pollution, but also significantly promote high-quality economic development. As far as carbon emissions are concerned, the construction of low carbon cities represents the governance level of cities in terms of low carbon. During the construction of low carbon cities, local governments have explored their own low-carbon development path, improved the environmental governance level of local governments, and achieved the effect of improving government air quality. On the other hand, the digital economy has also improved the government’s supervision and monitoring capacity. The government’s monitoring of pollution discharge and data collection have become more effective, forming a systematic monitoring system, standardized monitoring methods and timely monitoring feedback.

**Hypothesis 1:**  *The development of the digital economy has promoted the reduction of urban carbon emission intensity and achieved green development.*

#### 2.2.1. Improvement of Human Capital Investment

The digital economy has effectively reduced the intensity of urban carbon emissions by promoting urban human capital investment. Human capital investment includes not only the investment in compulsory education, high school and university, but also the investment in vocational ability training. The improvement of human capital investment can significantly improve the level of human capital, which can effectively promote high-quality economic development. Li et al. [41] believed that the government should actively increase the investment in human capital, thus promoting the effective supply of human capital and achieving high-quality economic development.

On the one hand, the digital economy drives the improvement of human capital level by promoting the improvement of human capital investment, thus combining with artificial intelligence, big data, industrial robots and other technologies of the digital economy. High quality human capital can further contribute to the multiplier effect of human capital investment, and expand the positive role of the digital economy in environmental protection and environmental governance. On the other hand, green development or low-carbon development itself requires workers to reserve certain skills, knowledge and quality. If the level of human capital is low, it is difficult to adapt to the needs of green development, and the positive role of green development is also limited. The higher the level of human capital, the stronger the ability to have green jobs, and the more conducive to promoting green economic development. Therefore, the development of the digital economy has promoted the improvement of human capital investment, further accelerated workers’ mastering of new knowledge and technology, promoted the accumulation and improvement of human capital, and thus matched with green development. In addition, with China’s economic transformation and development, China’s economy is increasingly demanding green development. The higher the level of human capital, the easier it is to find jobs that match green jobs.

Therefore, the higher the investment in human capital, the higher the level of human capital. The stronger the role of the digital economy in ecological environment protection, the more conducive it is to reduce the intensity of urban carbon emissions and improve the level of urban ecological environment.

**Hypothesis 2:**  *The development of the digital economy further promotes the reduction of urban carbon emission intensity by increasing human capital investment.*

#### 2.2.2. Improvement of Green Innovation Level

Technological progress is the driving force for overcoming the middle-income trap [42], and also the key to achieving green economic development. Green innovation plays an important role in achieving “green water and green mountains are golden mountains and silver mountains”. Green innovation is the basis for achieving urban economic development and environmental protection, and helps to reduce urban carbon emission intensity. The digital economy is a new driving force to improve innovation level [43]. The digital economy can effectively reduce urban carbon emission intensity by promoting urban green innovation level.

The digital economy can improve the level of urban green innovation by improving the efficiency of resource allocation, reducing financing constraints and green knowledge spillovers, and thus reduce the intensity of urban carbon emissions. Firstly, the digital economy has improved the efficiency of resource allocation, which makes it possible to further enhance the level of green innovation. Compared with traditional innovation activities, green innovation faces greater uncertainty and requires higher financial support. The digital economy can improve the efficiency of resource allocation and improve the production efficiency of enterprises. It not only saves production costs, but also improves the retained earnings of enterprises, providing internal financial support for enterprises to actively carry out green innovation. Secondly, the development of the digital economy promotes the reduction of urban carbon emission intensity by reducing the constraint level of green innovation financing. The development of the digital economy can improve the probability of information collection of banks and other institutions, reduce the degree of information asymmetry between banks and enterprises, reduce the cost of information communication, improve the matching quality between banks and enterprises and other economic entities, thus further reducing the external financing constraints faced by enterprises in green innovation. The decline of external financing constraints of enterprises has promoted the improvement of green innovation level of enterprises. The improvement of urban green innovation level can effectively reduce the intensity of urban carbon emissions. Finally, the development of the digital economy accelerates the spillover of green knowledge among different cities through network externalities [44], and promotes the reduction of urban carbon emission intensity. The development of the digital economy provides more information and information exchange channels for cities, which promotes the spillover of green knowledge among cities and further enhance the green innovation level of different cities. Green innovation can change the development mode of cities relying on high input and high pollution into a development mode relying more on innovation input and output, thereby reducing the intensity of urban carbon emissions.

In addition, the higher the level of economic development, the higher the economic complexity. The increase of economic complexity further increases the difficulty of reasonable allocation of resources, which may lead to more serious problems of ineffective resource allocation. The development of the digital economy provides a more timely and broader path for resource allocation within and between cities through big data, cloud platforms and other information collection and exchange technologies. Therefore, the development of the digital economy provides a basis for the improvement of urban resource allocation efficiency, and the improvement of urban resource efficiency further provides space for urban green innovation.

**Hypothesis 3:**  *The digital economy is conducive to improving the level of urban green innovation, thereby promoting the reduction of urban carbon emission intensity.*

## 3. Variables, Data and Research Design

### 3.1. Variable Selection and Descriptive Statistics

Explained variable: urban carbon emission intensity (*CO*). In this paper, carbon dioxide emissions divided by real GDP are used as a measure of urban carbon emission intensity. For economies in transition, it is necessary to maintain economic growth and protect the ecological environment. Therefore, taking carbon emission intensity as the emission reduction indicator is based on the full consideration of China’s economic and social development.

Core explanatory variable: urban digital economy level (*Dig*). Based on the method of Zhao et al. [45], this paper constructs an indicator system to measure the development level of the digital economy in cities from the two levels of internet development and digital financial inclusion, and uses the entropy method to calculate the development index of urban digital economy. In addition, this paper also uses the urban digital economy index released by Tencent Research Institute to test the robustness of replacing core explanatory variables.

In order to more accurately evaluate the impact of urban digital economy on carbon emission intensity, the following control variables are added: population size (*Peo*), industrial structure (*Str*), human capital (*Stu*), financial development (*Mon*), and foreign direct investment (*FDI*). In addition, this paper uses the year fixed effect to control the variable that affects the urban carbon emission intensity over year, and also controls the urban fixed effect to alleviate the endogenous problem caused by the error of missing variables. The endogenous test part also further adds the provincial fixed effect. We further add the lag phase I of urban carbon emission intensity as the control variable into the regression equation to reduce the endogenous problem of the model caused by missing variables. The standard errors in the regression process of this paper are all clustered to the enterprise level (except for the panel Tobit model).

### 3.2. Sample Selection and Data Sources

The sample of urban panel data used in this paper is from the Statistical Yearbook of Chinese Cities from 2012 to 2020, which excludes prefecture-level cities in the Tibet Autonomous Region, Hong Kong Special Administrative Region, Macao Special Administrative Region and Taiwan Province, and deletes the prefecture-level cities established and cancelled in 2013 and later, totaling 278 prefecture-level cities and above. In the robustness test, the digital economy index calculated by replacing the entropy method with the urban digital economy index of Tencent Research Institute is used for regression. The time range of the urban digital economy index of Tencent Research Institute is from 2015 to 2019. Descriptive statistical results of variables are shown in Table 1.

### 3.3. Basic Regression Model Setting

This paper mainly studies the impact of digital economy development on urban carbon emission intensity. In addition to adding control variables, it also controls the fixed effects of time (year) and region (city). The regression model is as follows:

(1) $$CO_{i,t}=\alpha +\beta Dig_{i,t}+\varphi X_{i,t}+\gamma_{t}+\delta_{i}+\varepsilon_{i,t}$$

In Equation (1), $CO_{i,t}$ are the explained variables in this paper, representing the carbon emission intensity of the *i* city in year *t*. $Dig_{i,t}$ is the key explanatory variable of this paper, representing the development level of the digital economy of city *i* in year *t*. *β* is the coefficient that we are most concerned about, indicating the impact of the development of the urban digital economy on the carbon emission intensity. If the regression result is significantly positive, it indicates that the development of the urban digital economy has increased the carbon emission intensity. If the regression result is significantly negative, it indicates that the development of the urban digital economy has reduced the carbon emission intensity. $X_{i,t}$ is the control variable, specifically: population size (*Peo*), industrial structure (*Str*), human capital (*Stu*), financial development (*Mon*), foreign direct investment (*FDI*). *α* is a constant term. $\varepsilon_{i,t}$ is the random error term. $\gamma_{t}$ and $\delta_{i}$ are year and city fixed effects, respectively.

## 4. Basic Regression Results

This paper first empirically studies the impact of the development of the urban digital economy on carbon emission intensity, and then conducts a robustness test from four aspects to verify the reliability of the basic regression results, followed by an endogenous test.

### 4.1. Analysis of Basic Results

The research on the impact of digital economy development on urban carbon emission intensity takes Equation (1) as the empirical research model, and the basic regression results are shown in Table 2. The explained variable is the carbon emission intensity of cities, and the core explanatory variable is the digital economy development level of cities at prefecture level and above. Model 1 in column (1) of Table 2 does not add control variables and the fixed effects of year and city. The regression coefficient of the digital economy is −0.4138, passing the test at the 1% significance level. The regression results show that the development of the digital economy has significantly promoted the reduction of urban carbon emission intensity. The second model in column (2) of Table 2 is based on model 1, adding year and urban fixed effects. The regression coefficient of the digital economy is −1.1451, passing the test at the 1% significance level. We are most concerned about the regression results of model 3 in column (3) of Table 2. Model 3 adds control variables on the basis of model 2, and controls the year and urban fixed effects. The regression coefficient of the digital economy is −0.3780, passing the test at the 5% significance level. The regression results show that the development of the digital economy has significantly promoted the reduction of urban carbon emission intensity.

The regression results from column (1) to (3) show that, under the condition that other conditions remain unchanged, the development of the digital economy has significantly promoted the decline of urban carbon emission intensity, and the development of the digital economy has an important role in promoting the green transformation of urban economy. Hypothesis 1 of this paper has been verified. The research conclusions of this paper are similar to those of Zhu et al. [46], which promote the sustainable development of the environment [47].

### 4.2. Robustness Test

The test results in Table 2 show that the development of the digital economy can effectively promote the reduction of urban carbon emission intensity. However, the robustness of the basic regression results has not been tested. This paper further verifies the robustness of the basic regression results by transforming core explanatory variables, deleting municipal samples, using panel Tobit model, and censoring and shrinking tail tests. In the process of robustness test based on Equation (1), the control variables, year and urban fixed effects are added, and the clustering robustness standard error at the urban level is adopted.

#### 4.2.1. Change the Measurement Method of Core Explanatory Variables

In the basic regression process of this paper, the core explanatory variable adopts the entropy method to measure the development level of the urban digital economy. In order to verify the robustness of the basic regression results, this paper uses the urban digital economy index released by Tencent Research Institute as the core explanatory variable for regression again. The regression results are shown in column (1) of Table 3. The regression coefficient of the digital economy is −0.0095. The test at the 10% significance level shows that the development of the digital economy has significantly reduced the intensity of urban carbon emissions. The basic regression results in Table 2 do not change due to the change in the measurement of core explanatory variables, and the basic conclusions are robust.

In addition, this paper also uses the number of internet users as a variable to measure the development level of the urban digital economy, and puts it into the regression equation to test again. The regression results are shown in in the regression results in column (2) of Table 3, the regression coefficient of the digital economy is −0.0409, passing the test at the 10% significance level. It still shows that the development of the digital economy can reduce the carbon emission intensity of cities. The basic regression results are robust.

#### 4.2.2. Delete Samples from Municipalities Directly under the Central Government

Considering that the administrative levels of municipalities and provinces (autonomous regions) are the same, this paper further deletes the samples of municipalities for another regression. The regression results of sample deletion of municipalities are shown in Table 3. The regression in column (3) of Table 3 shows that the regression coefficient of the digital economy is −0.3660, which passes the test at the significance level of 10%. The digital economy promotes the reduction of urban carbon emission reduction intensity, and there is no different result due to the change in sample selection. The basic regression result is reliable.

#### 4.2.3. Panel Tobit Model Regression

Because the urban carbon emission intensity data in this paper has the left deletion problem with 0 as the critical point, this paper further uses the panel Tobit model for regression. The regression results are shown in column (4) of Table 3. The regression coefficient of the digital economy is −0.0961, which passes the test at the significance level of 5%. The regression results still show that the development of the digital economy has promoted the decline in urban carbon emission intensity. The conclusions of this paper could not change due to the transformation of regression methods, so the basic regression results are robust.

#### 4.2.4. Shrinking and Ending Inspection

Due to measurement error and other reasons, the sample data could be biased to a certain extent, thus affecting the objectivity and accuracy of the regression results. Therefore, for the possible extreme values and other special cases, the sample can generally be shrunk and truncated. In this paper, we choose 1.5 and 97.5 percentiles for sample shrinkage, and lower than the 1st percentile and higher than the 99th percentile for sample truncation. The regression results are shown in Table 3. The column (5) of Table 3 is the regression result of the shrinkage test. The regression coefficient of the digital economy is −0.2915, which passes the test at the 1% significance level. The column (6) of Table 3 is the regression result of the truncation test. The regression coefficient of the digital economy is −0.2915, which also passes the test at the 1% significance level. The results of tail shrinking and censoring tests show that the basic regression results of this paper are reliable, and the development of the digital economy has significantly reduced the intensity of urban carbon emissions. 

### 4.3. Endogenetic Test

In this paper, the endogeneity test is conducted from two aspects: sample selectivity bias and missing variables.

#### 4.3.1. Sample Selection Deviation Test

Because different cities have different stages of economic development and different pillar industries, not all cities’ carbon emission intensity could decline, so this paper further adopts Heckman two−step method to test. Specifically, the Heckman two-step method uses the Probit model to calculate the inverse Mills ratio (IMRatio) in the first step, and brings the calculated inverse Mills ratio into the basic regression equation for re regression in the second step. In the first step of calculation, this paper calculates the growth rate of carbon emission intensity of each city. If the growth rate is greater than zero, it is taken as 1; if it is less than zero, it is taken as 0. Three regression methods are used in the Heckman two-step method in this paper.

The first way is to select the control variables in the basic regression as the influence variables of the first stage Probit model in the sample selection model. The test results of Heckman two-step method in this way are shown in column (1) of Table 4. The regression coefficient of the digital economy is −0.4613, which passes the test at the 5% significance level. The estimated coefficient of inverse Mills ratio is 6.6193, which fails the significance test. This indicates that the measurement process in this paper does not have the problem of sample selectivity error.

In the second way, only the lag period of carbon emission intensity is selected as the influence variable of the Probit model in the first stage of the sample selection model. The test results of Heckman two-step method in this way are shown in column (2) of Table 4. The regression coefficient of the digital economy is −0.3422, which passes the test at the 10% significance level. The estimated coefficient of inverse Mills ratio is −1.8977, which fails the significance test. This indicates that there is no sample selectivity error in the measurement process in this paper.

The third method is the integration of the first and second methods. It not only adds all control variables, but also adds the lag phase I of carbon emission intensity as the impact variable of the first phase Probit model in the sample selection model. The test results of Heckman two-step method in this way are shown in column (3) of Table 4. The regression coefficient of the digital economy is −0.3653, which passes the test at the 5% significance level. The estimation coefficient of inverse Mills ratio is −0.8665, which fails the significance test. This indicates that there is no sample selectivity error in the measurement process in this paper.

In conclusion, the Heckman two-step method shows that the regression samples in this paper cannot have the problem of selective bias, and the development of the digital economy can promote the reduction of urban carbon emission intensity.

#### 4.3.2. Missing Variables

In this paper, the control variables at the city level and the fixed effects of city and year are added to the basic regression, which reduces the endogenous problems caused by missing variables to a certain extent. However, there may still be missing variables due to time and regional changes in different regions. Therefore, this paper further adds provincial fixed effects and urban carbon emission intensity lags into the regression equation to further reduce the endogenous problems caused by missing variables. Specifically, column (4) of Table 4 adds provincial fixed effects, and the regression coefficient of the digital economy is −0.3780. It passes the test at the 5% significance level, indicating that after adding provincial fixed effects, the digital economy still significantly reduces the intensity of urban carbon emissions. In column (5) of Table 4, the lag phase I of urban carbon emission intensity is added as the control variable for regression again, without controlling the fixed effect of provinces. The regression results show that the regression coefficient of the digital economy is −0.4144, passing the test at the significance level of 5%, which still shows that the digital economy has promoted the reduction of urban carbon emission intensity. In column (6) of Table 4, the lag phase of urban carbon emission intensity is added, and the fixed effect of provinces is controlled. In the regression results, the regression coefficient of the digital economy is −0.4144. Through the test at the significance level of 5%, the improvement of the development level of the digital economy can effectively reduce the urban carbon emission intensity. Therefore, the basic research conclusions of this paper are reliable.

## 5. Mechanism Inspection

Through basic regression, robustness test and endogenous test, this paper has confirmed that the development of the digital economy can promote the reduction of urban carbon emission intensity, but how the digital economy plays this role is still worth further exploring. The regression equation of mechanism research in this paper is defined in Equation (2):

(2) $$Jizhi_{i,t}=\alpha +\mu Dig_{i,t}+\varphi X_{i,t}+\gamma_{t}+\delta_{i}+\varepsilon_{i,t}$$

In Equation (2), $Jizhi_{i,t}$ represents the mechanism variables in the regression analysis of this paper, which are the human capital investment and green innovation level of city *i* in year *t,* respectively. *μ* is the coefficient that we are most concerned about, and the other variables are the same as Equation (1). Human capital investment is expressed in urban education expenditure. The level of urban green innovation is expressed by urban per capita green patent data. Urban green patent data include green inventions and green utility models. The education expenditure is from the China Urban Statistical Yearbook from 2012 to 2020, and the urban green patent data is from CNRDS. In the regression process, all control variables are added and the city and year effects are fixed, and the standard error is clustered to the city level.

### 5.1. Improvement of Human Capital Investment

Firstly, this paper discusses the human capital investment mechanism that the digital economy promotes the reduction of urban carbon emission intensity. According to Equation (2), this paper uses human capital investment to regress digital economic variables in empirical analysis. The test results of human capital investment enhancement mechanism are shown in column (1) of Table 5. The regression coefficient of the digital economy is 0.4715, which passes the test at the significance level of 1%. It shows that the development of the digital economy has significantly promoted the improvement of urban human capital investment, and then led to the reduction of urban carbon emission intensity. Hypothesis 2 is validated.

### 5.2. Improve the Level of Green Innovation

The test results of the mechanism for improving the level of green innovation are shown in column (2) of Table 5. The regression coefficient of the digital economy is 1.9280, passing the test at the significance level of 1%. It shows that the development of the digital economy has significantly improved the level of urban green innovation, and the improvement of the level of green innovation has promoted the decline of urban carbon emission intensity. The higher the level of green innovation, the lower the intensity of urban carbon emissions. The level of green innovation is an important factor affecting the intensity of carbon emissions. The development of the digital economy can not only promote the improvement of urban green innovation level [48], but also promote the enhancement of enterprises’ green technology capability and the increase of enterprises’ green patents [49]. Hypothesis 3 of this paper is verified.

## 6. Further Analysis

This paper further analyzes the different classifications of urban location (eastern, central and western regions), urban administrative level, urban population size, resource-based cities and non-resource-based cities, with a view to studying the heterogeneity effect of digital economy development on urban carbon emission intensity and whether the mechanism of human capital investment and green innovation level is still in place. In the further analysis, all control variables are added based on Equations (1) and (2) to control the year and urban fixed effect, and the standard error of regression coefficient is clustered to the urban level.

### 6.1. East, Middle and West China

The regression results of the influence of urban digital economy development level on carbon emission intensity in the eastern, central and western regions are shown in Table 6. Column (1) lists the regression results of urban samples in the eastern region. The results show that the regression coefficient of the urban digital economy development level in the eastern region is −0.5800. The test at the 5% significance level shows that the improvement of the urban digital economy development level in the eastern region can effectively promote the reduction of the urban carbon emission intensity in the eastern region. Column (2) shows the regression results of the urban sample in the central region. The results show that the regression coefficient of the development level of the urban digital economy in the central region is −0.5182. The test at the 10% significance level shows that the development of the urban digital economy in the central region has significantly reduced the intensity of carbon emissions. Column (3) presents the regression results of the urban sample in the western region. The regression coefficient of the digital economy is 0.2273, which does not pass the significance test. To sum up, the regression results in Table 6 suggest that the development of the urban digital economy in eastern and central can promote the reduction of carbon emission intensity, but there is no significant effect in western China.

The possible reasons are that on the one hand, compared with the western region, the eastern and central regions have a higher level of urban economic development, and the eastern and central regions have a higher degree of marketization, which is more conducive to the formation and development of the digital economy. The industrial agglomeration and economies of scale produced by the formation and development of the digital economy are more significant, and the development of the digital economy is more conducive to the improvement of the efficiency of urban resource allocation. On the other hand, the cities in the eastern and central regions have relatively early economic transformation and upgrading, while the cities in the western regions have relatively lagged behind in transformation and upgrading. The regions with early economic transformation have started to lay out the infrastructure that is conducive to the development of the digital economy, and the digital infrastructure has formed economies of scale and played an economic role, while the regions with late transformation have limited the role of the digital economy.

Table 7 shows the test results of the influence mechanism of urban digital economy development level on carbon emission intensity in the eastern and central regions. Column (2) reports the regression results of the human capital investment mechanism of urban samples in the eastern region. The results show that the regression coefficient of the urban digital economy development level in the eastern region is 0.3796, passing the test at the significance level of 5%, indicating that the development of the digital economy has promoted the improvement of human capital investment in the eastern region, thereby reducing the carbon emission intensity of cities in the eastern region. Column (2) lists the regression results of the green innovation level mechanism of urban samples in the eastern region. The results show that the regression coefficient of the urban digital economy development level in the eastern region is 1.2854, passing the test at the 5% significance level, indicating that the development of the digital economy has promoted the improvement of the green innovation level in the eastern region, thereby reducing the carbon emission intensity of cities in the eastern region. Column (3) presents the regression results of the human capital investment mechanism of the city samples in the central region. The results show that the regression coefficient of the urban digital economy development level in the central region is 0.7794, passing the test at the 5% significance level, indicating that the urban digital economy development in the central region significantly reduces the carbon emission intensity through the increase of human capital investment. Column (4) shows the regression results of the green innovation level mechanism of urban samples in the central region. The results show that the regression coefficient of the urban digital economy development level in the central region is 1.3732, passing the test at the 10% significance level, indicating that the development of the urban digital economy in the central region has significantly reduced the carbon emission intensity by improving the green innovation level.

### 6.2. Urban Administrative Grade

Due to the different administrative levels among Chinese cities, the difference of resource gathering capacity is often caused. Cities at sub provincial level and above could receive more resource allocation, more policy preference and capital investment. Provincial capital cities and ordinary prefecture level cities gradually take the second place. In particular, ordinary prefecture level cities are often attracted by cities above the provincial capital level, and most of the resource-oriented cities or ordinary prefecture level cities are even more lacking in technology, capital and human capital.

Table 8 shows for the regression results of the impact of the urban digital economy development level of different administrative levels on carbon emission intensity. Column (1) lists the regression results of the sample of cities at and above the sub-provincial level. The results show that the regression coefficient of the development level of the digital economy in cities at and above the sub-provincial level is −0.2208. The test at the significance level of 5% shows that the development of the digital economy in cities at and above the sub-provincial level has reduced the intensity of urban carbon emissions. Column (2) shows the regression results of the provincial capital city sample. The results show that the regression coefficient of the provincial capital city’s digital economic development level is 0.2824, which fails the significance test. Column (3) displays the regression results of other cities. The regression coefficient of the digital economy is −0.4815, which fails the significance test. In short, the regression results in Table 8 show that China’s urban administrative level could affect the effect of digital economy development on urban carbon emission intensity. The development of the digital economy in cities at sub provincial level and above can significantly promote the reduction of urban carbon emission intensity, but the impact of digital economy development on carbon emission intensity in provincial capital cities and other cities does not show a significant effect.

Therefore, the impact of digital economy development on urban carbon emission intensity varies with the different administrative levels of cities. The possible reasons are that, on the one hand, cities at sub provincial level and above have more preferential policies and stronger strength, so they are the first to receive national policy support in the development of the digital economy, and for their own province or city, the government could also use stronger strength to support their first development of the digital economy. Therefore, the development level and stage of the digital economy are higher. On the other hand, the marketization level of cities at or above the sub provincial level is among the best in domestic cities. Both the government business environment and the market resource allocation capacity are stronger than other cities. Therefore, the development of the digital economy is more conducive to improving urban environmental performance. Therefore, the promotion effect of the digital economy on the reduction of carbon emission intensity in sub provincial and above cities is more obvious, and these cities have also obtained the positive effect of the digital economy on environmental governance earlier.

The sample mechanism test results of cities above sub-provincial level are also shown in Table 8. Columns (4) and (5) are the mechanism test results of the human capital investment and green innovation level of the cities above the sub provincial level. The results show that the regression coefficients of the digital economy of cities above the sub-provincial level are 0.6008 and 0.9113, respectively, which have passed the test at the significance level of 5% and 10%, respectively, indicating that in cities above the sub-provincial level, the development of the digital economy has promoted the increase of human capital investment and the improvement of the level of green innovation, thus reducing the intensity of carbon emissions.

### 6.3. Urban Population Size

In addition to the differences in location and administrative level, there are also differences in city size. We divide cities with a population of more than 5 million into big cities, medium-sized cities between 1 million and 5 million, and small cities less than 1 million. We further analyze the impact of the digital economy on carbon emission intensity in different cities. The regression results are shown in Table 9. Column (1) lists the regression results of the sample of large cities. The results show that the regression coefficient of the development level of the digital economy in the sample of large cities is −0.5603. The test at the significance level of 5% shows that the development of the digital economy can promote the reduction of carbon emission intensity in large cities. Column (2) shows the regression results of the sample of medium-sized cities. The regression coefficient of the digital economy of medium-sized cities is −0.0747, which failed the significance test. Column (3) presents the regression results of small city samples. The regression coefficient of the digital economy is 2.0203, which fails the significance test. In a word, the regression results in Table 9 show that the development of the digital economy in large cities has promoted the reduction of carbon emission intensity, but in the sample of small and medium-sized cities, the development of the digital economy has not promoted the reduction of urban carbon emission intensity.

Table 9 shows the mechanism test results of the sample of large cities. Columns (4) and (5) are the mechanism test results of human capital investment and green innovation level of large cities, respectively. The results show that the regression coefficients of the digital economy in big cities are 0.5435 and 2.2792, respectively, which pass the test at the significance level of 1%. It shows that the development of the digital economy in big cities has promoted the increase of their human capital investment and the improvement of their green innovation level, thereby reducing their carbon emission intensity.

### 6.4. Resource-Based Cities and Non-Resource-Based Cities

The transformation and development of resource-based cities has played a vital role in the healthy development of China’s economy and society. Most resource-based cities take the mining and processing of mineral resources with high pollution and high energy consumption as their pillar industries. As early as 2007, the State Council issued Several Opinions of the State Council on Promoting the Sustainable Development of Resource based Cities, which defined that resource-based cities should strengthen environmental politics and ecological protection. However, no matter the economic and social transformation [50] and development of resource-based cities, or the ecological environment protection, the effect is not obvious. Industrial upgrading is an important means to balance economic development and environmental protection in resource-based cities. The development of the digital economy may provide an effective way for resource-based cities to achieve the unification of “green water and green mountains” and “golden mountains and silver mountains”, and open the key links of industrial upgrading of resource-based cities.

Table 10 shows the regression results of the impact of the development level of the digital economy of resource-based cities and non-resource-based cities on carbon emission intensity. Column (1) of Table 10 shows the regression results of the sample of resource-based cities. The regression coefficient of the development level of the digital economy is −0.3225, which fails the significance test. Column (2) of Table 10 shows the regression results of the sample of non-resource cities. The regression coefficient of the digital economy is −0.3632, which passes the test at the significance level of 5%. Therefore, the digital economy promotes the decline of urban carbon emission intensity and plays a more significant role in non-resource cities. The test results of carbon emission reduction mechanism for non-resource-based cities are shown in columns (3)–(4) of Table 10. Columns (3) and (4) of Table 10 are the mechanism tests of human capital investment and green innovation level, respectively. The regression coefficients of the digital economy in the regression results are 0.3329 and 1.6160, respectively, passing the test at 5% and 1% significance levels. It shows that in non-resource cities, the development of the digital economy promotes the reduction of urban carbon emission intensity by increasing human capital investment and improving the level of green innovation.

For resource-based cities and non-resource-based cities, the development of the digital economy in non-resource-based cities can more effectively reduce carbon emission intensity. China’s resource-based cities have different classifications. The National Sustainable Development Plan for Resource-based Cities (2013–2020) divides resource-based cities into four types: growth, maturity, recession and regeneration. Does the digital economy not promote the reduction of urban carbon emission intensity among the four types of resource-based cities? It is worth further study.

Regression results of the impact of the digital economy on urban carbon emission intensity in four different types of resource-based cities are shown in Table 11. Column (1) shows the regression results of the sample of growth resource-based cities. The regression coefficient of the digital economy development level is 0.8214, which fails the significance test. Column (2) reports the regression results of the sample of mature resource-based cities. The regression coefficient of the digital economy development level is 1.0426, which fails the significance test. Column (3) displays the regression results of the sample of declining resource-based cities. The regression coefficient of the digital economy development level is 0.6427, which fails the significance test. Column (4) shows the regression results of the sample of renewable resource-based cities. The regression coefficient of the digital economy is −0.6057, which passes the test at the level of 10% significance. To sum up, the digital economy promotes the reduction of urban carbon emission intensity, and plays a more significant role in renewable cities among non-resource-based cities. Therefore, in Table 10, the effect of the digital economy on the reduction of carbon emission intensity of resource-based cities is not significant, which is mainly due to the fact that the effect is not significant in growing, mature and declining resource-based cities.

In China’s resource-based cities, they are mainly cities that are gradually established and emerging relying on the mining of coal, oil, forests and non-ferrous metal minerals. According to the different resources that resource-based cities mainly rely on, this paper divides resource-based cities into coal resource-based cities, oil resource-based cities, iron ore resource-based cities, forest resource-based cities and other mineral resource-based cities. To further explore the impact of the digital economy on carbon emissions of different resource-based cities.

Table 12 shows the regression results of the impact of digital economy development level of different resource-oriented cities on carbon emission intensity. Column (1) shows the resource-based cities dominated by iron ore. The regression coefficient of the digital economy is −32.6671. Through the test at the significance level of 5%, it is shown that the development of the digital economy in resource-based cities dominated by iron ore has promoted the decline of urban carbon emission intensity. Column (2) is a resource-based city dominated by oil. The regression coefficient of the digital economy is −2.2687, passing the test at the 10% significance level, indicating that the development of the digital economy in resource-based cities dominated by oil also significantly promoted the reduction of urban carbon emission intensity. Columns (3)–(5) are resource-based cities dominated by coal, forest and other minerals. The regression coefficients of the digital economy are 2.1096, −0.1779 and 1.3966, respectively, which have not passed the significance test, indicating that the carbon intensity decline effect of urban digital economy development dominated by these three resources has not yet appeared. To sum up, the digital economy has significantly reduced the urban carbon emission intensity for resource-based cities dominated by iron ore and oil. In resource-based cities dominated by coal, forest and other minerals, the reduction of carbon emission intensity has not had a significant effect. Therefore, the digital economy of resource-based cities in Table 10 does not significantly promote the reduction of urban carbon emission intensity, which is more caused by the non-significant reduction effect of carbon emission intensity of coal, forest and other mineral resource-based cities.

## 7. Conclusions 

It is worth exploring whether the development of the digital economy can promote the protection and improvement of the ecological environment, and whether it can realize that “green water and green mountains are golden mountains and silver mountains”. To some extent, the reduction of carbon emission intensity can indicate that China has achieved the protection of ecological environment in the process of economic development. Therefore, this paper analyzes its impact on the decline of urban carbon emission intensity from the perspective of the digital economy. This paper first analyzes the theoretical basis and impact mechanism of the digital economy on urban carbon emission intensity, and then empirically analyzes the impact of the digital economy on urban carbon emission intensity and its mechanism using 278 city-level statistical data from 2011 to 2019. The research conclusions are as follows.

First, the development of the urban digital economy has significantly promoted the reduction of urban carbon emission intensity. In the process of the development of the digital economy, the production efficiency and government governance capacity have been improved, and the intensity of urban carbon emissions has decreased. After changing core explanatory variables, deleting municipality samples, using the panel Tobit model, and censoring and shrinking tail tests, the conclusion is still robust. Second, in terms of impact mechanism, the development of the urban digital economy, on the one hand, promotes the increase of urban human capital investment level, on the other hand, promotes the improvement of urban green innovation level, thereby reducing the intensity of urban carbon emissions. Third, the development of the urban digital economy has significantly promoted the reduction of carbon emission intensity in cities in the eastern and central regions, cities above the sub provincial level, large cities and non-resource-based cities. As far as resource-based cities are concerned, in renewable resource-based cities and resource-based cities dominated by iron ore and oil mining, the effect of the digital economy on the reduction of urban carbon emission intensity is more obvious.

## 8. Policy Recommendations

Accordingly, the following policy recommendations are proposed. First, we should promote the development level of the urban digital economy. The development of the digital economy has promoted the reduction of urban carbon emission intensity. It shows that the development of the digital economy plays a positive role in ecological environment protection. Therefore, on the one hand, local governments should actively improve the infrastructure of the digital economy to lay a solid foundation for the development of the digital economy. On the other hand, entrepreneurs should give full play to their entrepreneurial spirit, promote the digital transformation of enterprises, and promote the development of the digital economy and carbon emission reduction at the enterprise level. Second, we could achieve the improvement of human capital investment and green innovation capability. The development of the digital economy has reduced the intensity of urban carbon emissions by promoting the level of urban human capital investment and green innovation. Therefore, local governments and enterprises should actively increase investment in human capital to make it more suitable for the development of the digital economy. Local governments and enterprises should also increase investment in R&D, especially in green innovation, to promote the improvement of production efficiency, ease the internal and external financing constraints of green innovation, and promote the improvement of green innovation level. Third, we should achieve balanced development of the digital economy. It can be seen from the empirical study that the carbon emission intensity reduction effect of the digital economy is more obvious in cities in the eastern and central regions, sub-provincial cities, large cities and non-resource-based cities. It shows that the development level of China’s digital economy is not balanced. Therefore, on the one hand, local governments and enterprises should give full play to their own role to realize the development of the digital economy. On the other hand, they should give sufficient policy and financial support, and use the big data national strategy and the national computing network to achieve the balanced development of the digital economy.

## Figures and Tables

**Table 1:** Variable definition and descriptive statistical results.

Variable Type	Variable Definition	Variable Definition	Mean Value	Standard Deviation
Interpreted variable	Carbon emission intensity (*CO*)	Urban carbon emissions/urban GDP	0.4605	1.8569
Explanatory variable	Digital Economy (*Dig*)	Development level of urban digital economy	0.0949	0.0553
Control variable	Population size (*Peo*)	Total urban population at the end of the year	5.8874	0.6975
	Industrial structure (*Str*)	Output value of secondary industry/urban GDP	3.8190	0.2590
	Human capital (*Stu*)	Number of students in primary and secondary schools	3.3635	11.2038
	Financial Development (*Mon*)	Loan balance of financial institutions at the end of the year	3199.5065	6647.4130
	Foreign Direct Investment (*FDI*)	Actual utilization of foreign direct investment	10.0947	1.8665

Source: the data is collected by the author.

**Table 2:** Basic regression results.

Explained Variable: Urban Carbon Emission Intensity	(1)	(2)	(3)
	Model I	Model II	Model III
Digital economy	−0.4138 ***	−1.1451 ***	−0.3780 **
	(0.1591)	(0.3620)	(0.1799)
Constant	0.4212 ***	0.0617	2.5501 **
	(0.0152)	(0.5187)	(1.0373)
Control variable	No	No	Yes
Year fixed effect	No	Yes	Yes
Urban fixed effect	No	Yes	Yes
Obs.	2395	2395	2395
Within R^2^	0.3686	0.0587	0.3693
Number of cities	278	278	278

Notes: ***, ** are significant at the level of 1% and 5%, respectively. The values in parentheses are the standard errors of variable estimation coefficients clustered by cities.

**Table 3:** Results of robustness test.

Explained Variable: Urban Carbon Emission Intensity	(1)	(2)	(3)	(4)	(5)	(6)
	Tencent Digital Economic Index	Number of Internet Users	Eliminate Municipality Directly under the Central Government	Tobit Model	Shrinkage Test	Censoring Test
Digital Economy a	−0.0095 *					
	(0.0050)					
Digital Economy b		−0.0409 *				
		(0.0240)				
Digital economy			−0.3660 *	−0.0961 **	−0.2915 ***	−0.2915 ***
			(0.1999)	(0.0490)	(0.1087)	(0.1002)
Control variable	Yes	Yes	Yes	Yes	Yes	Yes
Year fixed effect	Yes	Yes	Yes	Yes	Yes	Yes
Urban fixed effect	Yes	Yes	Yes	Yes	Yes	Yes
Constant	0.2083	3.1014 ***	2.5915 **	4.8172 ***	2.5759 ***	2.8754 ***
	(2.9518)	(1.1255)	(1.0403)	(1.2940)	(0.6345)	(0.5841)
Obs.	1037	2390	2359	2395	2395	2283
Within R^2^	0.394	0.3697	0.3694	−	0.8267	0.8359

Notes: ***, **, * are significant at the level of 1%, 5% and 10%, respectively. The values in parentheses are the standard errors of variable estimation coefficients clustered by cities. Digital Economy a is the urban digital economy index released by Tencent Research Institute. Digital Economy b is the number of internet users.

**Table 4:** Endogenous test results.

	(1)	(2)	(3)	(4)	(5)	(6)
Digital economy	−0.4613 **	−0.3422 *	−0.3653 **	−0.3780 **	−0.4144 **	−0.4144 **
	(0.2203)	(0.1768)	(0.1737)	(0.1810)	(0.2018)	(0.2032)
*IMRatio*	6.6193	−1.8977	−0.8665			
	(5.8118)	(1.3038)	(0.6366)			
Constant	8.8624	4.4803 ***	1.6815	2.5501 **	2.8825 **	2.8825 **
	(5.7697)	(1.3132)	(1.4472)	(1.0437)	(1.2135)	(1.2219)
Control variable	Yes	Yes	Yes	Yes	Yes	Yes
Year fixed effect	Yes	Yes	Yes	Yes	Yes	Yes
Urban fixed effect	Yes	Yes	Yes	Yes	Yes	Yes
Provincial fixed effect	Yes	Yes	Yes	Yes	No	Yes
Obs.	2392	2122	2122	2395	2124	2124
Within R^2^	0.0020	0.0026	0.0025	0.3693	0.3653	0.3653

Notes: ***, **, * are significant at the level of 1%, 5% and 10%, respectively. The values in parentheses are the standard errors of variable estimation coefficients clustered by cities.

**Table 5:** Mechanism inspection results.

	(1)	(2)
	Human Capital Investment	Green Innovation Level
Digital economy	0.4715 ***	1.9280 ***
	(0.1535)	(0.5331)
Constant	5.6600 ***	−2.3434
	(1.0044)	(1.6156)
Control variable	Yes	Yes
Year fixed effect	Yes	Yes
Urban fixed effect	Yes	Yes
Obs.	2395	2389
Within R^2^	0.1960	0.2177

Notes: *** is significant at the level of 1%. The values in parentheses are the standard errors of variable estimation coefficients clustered by cities.

**Table 6:** Regression results of urban samples in eastern, central and western China.

	(1)	(2)	(3)
	Eastern Region	Central Region	Western Region
Digital economy	−0.5800 **	−0.5182 *	0.2273
	(0.2912)	(0.2864)	(0.5303)
Constant	9.6363 **	1.8999 ***	−0.6146
	(4.0435)	(0.5752)	(1.9841)
Control variable	Yes	Yes	Yes
Year fixed effect	Yes	Yes	Yes
Urban fixed effect	Yes	Yes	Yes
Obs.	881	868	646
Within R^2^	0.2349	0.8781	0.9147

Notes: ***, **, * are significant at the level of 1%, 5% and 10%, respectively. The values in parentheses are the standard errors of variable estimation coefficients clustered by cities. The explained variables are the urban carbon emission intensity.

**Table 7:** Mechanism test results of east and central China.

	(1)	(2)	(3)	(4)
	Eastern Region		Central Region	
	Human Capital Investment	Green Innovation Level	Human Capital Investment	Green Innovation Level
Digital economy	0.3796 **	1.2854 **	0.7794 **	1.3732 *
	(0.1461)	(0.4935)	(0.3559)	(0.7392)
Constant	3.5412 *	−12.9212 ***	6.6639 ***	−0.0839
	(1.8263)	(3.0088)	(1.1598)	(0.4155)
Control variable	Yes	Yes	Yes	Yes
Year fixed effect	Yes	Yes	Yes	Yes
Urban fixed effect	Yes	Yes	Yes	Yes
Obs.	881	880	868	866
Within R^2^	0.2536	0.3142	0.2402	0.1747

Notes: ***, **, * are significant at the level of 1%, 5% and 10%, respectively. The values in parentheses are the standard errors of variable estimation coefficients clustered by cities.

**Table 8:** Regression results of samples of cities with different administrative levels.

	(1)	(2)	(3)	(4)	(5)
	Cities above Sub Provincial Level	Provincial Capital	Other Cities	Cities above Sub Provincial Level	
				Human Capital Investment	Green Innovation Level
Digital economy	−0.2208 **	0.2824	−0.4815	0.6008 **	0.9113 *
	(0.1018)	(0.4704)	(0.3373)	(0.2601)	(0.4721)
Constant	3.7417	4.2284	2.4147 **	−5.5308	−11.9261
	(2.3203)	(6.0485)	(1.1468)	(3.6063)	(7.0629)
Control variable	Yes	Yes	Yes	Yes	Yes
Year fixed effect	Yes	Yes	Yes	Yes	Yes
Urban fixed effect	Yes	Yes	Yes	Yes	Yes
Obs.	159	132	2104	159	159
Within R^2^	0.8543	0.4677	0.3664	0.5266	0.0935

Notes: **, * are significant at the level of 5% and 10%, respectively. The values in parentheses are the standard errors of variable estimation coefficients clustered by cities.

**Table 9:** Regression results of large, medium and small cities.

Explained Variable: Urban Carbon Emission Intensity	(1)	(2)	(3)	(4)	(5)
	Big City	Medium-Sized City	Small City	Big City	
				Human Capital Investment	Green Innovation Level
Digital economy	−0.5603 **	−0.0747	2.0203	0.5435 ***	2.2792 ***
	(0.2767)	(0.1544)	(2.0893)	(0.1961)	(0.7123)
Constant	−0.2217	4.6597 ***	2.354	6.7183 ***	−5.1531 ***
	(3.2434)	(1.5960)	(5.2189)	(1.5780)	(1.8703)
Control variable	Yes	Yes	Yes	Yes	Yes
Year fixed effect	Yes	Yes	Yes	Yes	Yes
Urban fixed effect	Yes	Yes	Yes	Yes	Yes
Obs.	907	1418	70	905	903
Within R^2^	0.1952	0.6198	0.9376	0.1096	0.2821

Notes: ***, ** are significant at the level of 1% and 5%, respectively. The values in parentheses are the standard errors of variable estimation coefficients clustered by cities.

**Table 10:** Regression results of resource-based cities and non-resource-based cities.

	(1)	(2)	(3)	(4)
	Resource-Based City	Non-Resource-Based Cities	Non-Resource-Based Cities	
			Human Capital Investment	Green Innovation Level
Digital economy	−0.3225	−0.3632 **	0.3329 **	1.6160 ***
	(0.8891)	(0.1731)	(0.1320)	(0.5436)
Constant	2.1620 **	3.0900 *	6.2054 ***	−4.8307 **
	(1.0745)	(1.8421)	(1.0627)	(2.3030)
Control variable	Yes	Yes	Yes	Yes
Year fixed effect	Yes	Yes	Yes	Yes
Urban fixed effect	Yes	Yes	Yes	Yes
Obs.	943	1452	1452	1449
Within R^2^	0.3362	0.4466	0.1883	0.2635

Notes: ***, ** are significant at the level of 1% and 5%, respectively. The values in parentheses are the standard errors of variable estimation coefficients clustered by cities.

**Table 11:** Regression results of samples of resource-based cities and non-resource-based cities.

	(1)	(2)	(3)	(4)
	Growth	Mature Type	Declination Type	Regenerative Type
Digital economy	0.8214	1.0426	0.6427	−0.6057 *
	(0.5608)	(1.1299)	(0.8919)	(0.3424)
Constant	7.1617 *	−1.0175	4.2807	31.0696 **
	(3.8404)	(3.4389)	(2.8872)	(14.2426)
Control variable	Yes	Yes	Yes	Yes
Year fixed effect	Yes	Yes	Yes	Yes
Urban fixed effect	Yes	Yes	Yes	Yes
Obs.	99	523	190	131
Within R^2^	0.9219	0.3105	0.9240	0.7429

Notes: **, * are significant at the level of 5% and 10%, respectively. The values in parentheses are the standard errors of variable estimation coefficients clustered by cities.

**Table 12:** Regression results of samples of resource-based cities dominated by different industries.

	(1)	(2)	(3)	(4)	(5)
	Iron Ore	Petroleum	Coal	Forest	Other Minerals
Digital economy	−32.6671 **	−2.2687 ***	2.1096	−0.1779	1.3966
	(13.2917)	(0.5239)	(1.9016)	(0.5792)	(0.9907)
Constant	−9.5631	4.7608 **	1.1803	4.8001	1.6408
	(21.9625)	(2.0404)	(3.2374)	(2.9640)	(2.9201)
Control variable	Yes	Yes	Yes	Yes	Yes
Year fixed effect	Yes	Yes	Yes	Yes	Yes
Urban fixed effect	Yes	Yes	Yes	Yes	Yes
Obs.	81	75	360	47	225
Within R^2^	0.3090	0.9238	0.3567	0.9833	0.7894

Notes: ***, ** are significant at the level of 1% and 5%, respectively. The values in parentheses are the standard errors of variable estimation coefficients clustered by cities.

## Data Availability

The data presented in this study are openly available in statistical yearbooks and official websites.
